# Coding of Non-coding RNA: Insights Into the Regulatory Functions of Pri-MicroRNA-Encoded Peptides in Plants

**DOI:** 10.3389/fpls.2021.641351

**Published:** 2021-02-25

**Authors:** Yi Ren, Yue Song, Lipeng Zhang, Dinghan Guo, Juan He, Lei Wang, Shiren Song, Wenping Xu, Caixi Zhang, Amnon Lers, Chao Ma, Shiping Wang

**Affiliations:** ^1^Department of Plant Science, School of Agriculture and Biology, Shanghai Jiao Tong University, Shanghai, China; ^2^Department of Horticulture, College of Agriculture, Shihezi University, Shihezi, China; ^3^Department of Postharvest Science of Fresh Produce, Volcani Center, Agricultural Research Organization, Bet Dagan, Israel; ^4^Key Laboratory of Agro-products Processing Technology of Shandong, Institute of Agro-food Science and Technology, Shandong Academy of Agricultural Sciences, Jinan, China

**Keywords:** miPEP, miRNA-encoded peptide, miRNA, pri-miRNA, non-coding RNA, peptides

## Abstract

Peptides composed of a short chain of amino acids can play significant roles in plant growth, development, and stress responses. Most of these functional peptides are derived by either processing precursor proteins or direct translation of small open reading frames present in the genome and sometimes located in the untranslated region sequence of a messenger RNA. Generally, canonical peptides serve as local signal molecules mediating short- or long-distance intercellular communication. Also, they are commonly used as ligands perceived by an associated receptor, triggering cellular signaling transduction. In recent years, increasing pieces of evidence from studies in both plants and animals have revealed that peptides are also encoded by RNAs currently defined as non-coding RNAs (ncRNAs), including long ncRNAs, circular RNAs, and primary microRNAs. Primary microRNAs (miRNAs) have been reported to encode regulatory peptides in *Arabidopsis*, grapevine, soybean, and *Medicago*, called miRNA-encoded peptides (miPEPs). Remarkably, overexpression or exogenous applications of miPEPs specifically increase the expression level of their corresponding miRNAs by enhancing the transcription of the *MIRNA* (*MIR*) genes. Here, we first outline the current knowledge regarding the coding of putative ncRNAs. Notably, we review in detail the limited studies available regarding the translation of miPEPs and their relevant regulatory mechanisms. Furthermore, we discuss the potential cellular and molecular mechanisms in which miPEPs might be involved in plants and raise problems that needed to be solved.

## Introduction

For a long time, canonical phytohormones, such as auxin and cytokinin, offer the main perspective in our understanding of regulatory networks modulating plant growth, development, and stress response (Dubois et al., 2018; Kieber and Schaller, 2018). In the last decades, an increasing number of studies have focused on the central role of small peptides, called peptide-hormones, as short- or long-distance signaling molecules to integrate internal cues with external environment stimuli (Huffaker et al., 2011; Ohkubo et al., 2017; Nakaminami et al., 2018; Olsson et al., 2019). Peptides are typically composed of 2 to 100 amino acid residues (Tavormina et al., 2015) and are commonly secreted into the apoplast. Known peptides usually act as ligands that bind to their receptors to activate downstream signaling cascades involved in plant innate immunity (Huffaker et al., 2006; Yamaguchi et al., 2006; Huffaker and Ryan, 2007), nutrient homeostasis (Okamoto et al., 2016), reproduction process (Kachroo et al., 2001; Okuda et al., 2009; Qu et al., 2015), stress response (Nakaminami et al., 2018; Takahashi et al., 2018), and morphogenesis (Yamaguchi et al., 2016; Qian et al., 2018). Although the majority of the reported functional peptides are derived from the processing of precursor proteins or the coding of small open reading frames (sORFs), numerous pieces of evidence from plants and animals have suggested that previously annotated non-coding RNAs (ncRNAs) may encode peptides, expanding the peptidome complexity (Ruiz-Orera et al., 2014; Lauressergues et al., 2015; Legnini et al., 2017). Primary microRNAs (pri-miRNAs), which are transcribed from the *MIR* genes and subsequently processed to produce the mature miRNAs, can actually encode regulatory miRNA-encoded peptides (miPEPs) in plants (Wang L. and Wang, 2015; Lv et al., 2016; Julkowska, 2020). According to available results, overexpression or external application of miPEPs can positively regulate the mature miRNAs by enhancing the transcription of their associated *MIR* genes, which is similar to the innate immunity system in plants where the endogenous peptides can also increase expression of their encoding precursor genes (Huffaker and Ryan, 2007; Huffaker et al., 2011; Couzigou et al., 2016). In this review, we review the coding of ncRNA, emphatically, focusing on the translation of pri-miRNA and their relevant biological functions and possible regulatory mechanisms.

## Peptidome Complexity in Plants

**FIGURE 1:**
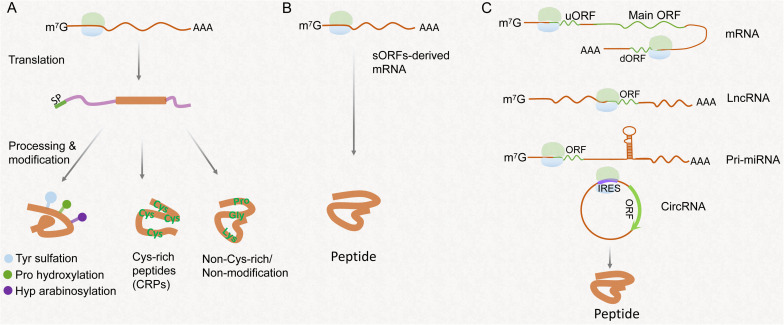
Schematic presentation of the different pathways of peptide synthesis and processing. **(A)** Peptides processed from the precursor containing signaling peptide (SP). Modifications of peptides contain Tyr sulfation, proline hydroxylation, and hydroxyproline arabinosylation. Cys-rich peptides usually contain 2–16 Cys residues. Non-Cys-rich/non-modificational peptides contain several key residues such as proline, Gly, and lysine responsible for biological activity. **(B)** Peptides encoded by sORFs in genome. **(C)** Peptides encoded by an upstream ORF (uORF) or downstream ORF (dORF) of a protein-coding region, non-coding RNAs, including lncRNAs, pri-miRNAs, and circRNAs.

Since systemin was first characterized in tomato, plant peptides are emerging as significant signaling molecules involved in different physiological processes (Pearce et al., 1991). They are categorized into precursor-derived peptides and non-precursor-derived peptides based on their biogenesis (Figure 1). The precursor-derived peptides are further classified into post-translationally modified peptides, cysteine-rich peptides (CRPs), and peptides without rich cysteine or post-translational modification (Figure 1A). The non-precursor-derived peptides are mainly encoded by sORFs hidden in the plant genome, additional short ORFs in messenger RNA (mRNA) transcripts, and so-called ncRNAs (Figures 1B,C; Hanada et al., 2013; Tavormina et al., 2015; Oh et al., 2018).

### Peptides Encoded by Conventional Open Reading Frames

The origins, functions, and functional mechanisms of peptides encoded by conventional ORFs in plants have been well-reviewed (Tavormina et al., 2015; Olsson et al., 2019). Generally, mature precursor-derived peptides are initially translated into larger non-functional prepropeptides and further processed by proteolytic cleavage and modification such as tyrosine (Tyr) sulfation, proline hydroxylation, and hydroxyproline arabinosylation, to yield biologically active peptides (Figure 1A; Matsubayashi, 2014; Olsson et al., 2019). The second precursor-derived peptides are the CRPs characterized by a domain with 2–16 cysteine residues (Matsubayashi, 2014). CRPs are also processed, and the typical intramolecular disulfide bonds are catalyzed by protein disulfide isomerases (Figure 1A; Tavormina et al., 2015; Olsson et al., 2019). The third group of peptides processed from the non-functional precursors is named “non-Cys-rich/non-modification peptides,” which contain several important amino acid residues such as proline, Gly, and lysine critical for biological activity (Figure 1A; Tavormina et al., 2015). Additionally, most gene annotation algorithms do not effectively distinguish between coding and non-coding sequences when the coding sequences are small. Therefore, thousands of sORFs are failed to be annotated in the plant genome as coding for proteins (Andrews and Rothnagel, 2014; Takahashi et al., 2019). In *Arabidopsis*, ∼8,000 putative sORFs with high coding potential are identified, of which ∼10% of identified peptides have a function based on the visible phenotypic effects revealed after their overexpression (Hanada et al., 2013). Therefore, it is a reasonable assumption that many functional sORFs are hidden in the plant genome (Hsu and Benfey, 2018; Figure 1B). In general, canonical peptides are thought to be phytohormone-like signaling molecules that mediate short- or long-distance intercellular communication and play an important role in regulating growth and development in plants (Ohkubo et al., 2017; Olsson et al., 2019).

### Coding of Short Open Reading Frames in Putative Non-coding RNAs

In eukaryotic mRNA, one or more short ORFs may exist in 5× leader sequence [or 5′ untranslated region (UTR)] located in the upstream of the main protein-coding ORF, called upstream open reading frame (uORF) (Chen J. et al., 2020; Kurihara, 2020; Figure 1C). The uORF presumably serves as a post-transcriptional *cis*-regulatory element that represses the transcription of main protein-coding ORF by causing ribosome stalling and nonsense-mediated decay (von Arnim et al., 2014). In one case, vitamin C/ascorbate content is determined by the GDP-L-galactose phosphorylase (GGP) enzyme. An uORF located in the upstream UTR of the *GGP* gene, encoding 60-a.a. length peptide, serves as a *cis*-acting element that represses the translation of the downstream *GGP* ORF under high ascorbate concentration (Laing et al., 2015). Editing the uORF of *GGP* increases the vitamin C content by ∼150% (Zhang et al., 2018). In *Arabidopsis*, AtHB1 belongs to the homeodomain-leucine zipper transcription factor family. The translation of *AtHB1* is post-transcriptionally repressed by the uORF located in the upstream of 5’ UTR of *AtHB1* through a ribosome stalling mechanism. This uORF encodes a conserved peptide in flowering plants, called CPuORF (Ribone et al., 2017; van der Horst et al., 2019). In addition to uORF in 5’ UTR, hundreds of sORFs have been identified in 3’ UTR, called downstream UTRs (dUTRs), by ribosome profiling sequencing and proteomics analyses in mammalian cell (Chen J. et al., 2020; Wu et al., 2020). Contrary to uORFs, dUTRs were described to enhance the translation of their corresponding main ORF (Wu et al., 2020). Whether translation of dUTRs occurs in plants remains to be shown. Although the translation of sORFs derived from the 5’ or 3’ UTR of mRNA has been investigated, the biological functions of such peptides are not fully understood yet.

Ribosome profiling sequencing provides a feasible method to explore the coding potential of putative ncRNAs such as long non-coding RNAs (lncRNAs), circular RNAs (circRNAs), and pri-miRNA, although this cue is not sufficient to classify transcripts as coding or non-coding (Ingolia et al., 2012; Guttman et al., 2013). Recently, ncEP, a manually curated database for collecting validated ncRNA-encoded proteins or peptides, is constructed and enriches the repository of coding RNAs (Liu et al., 2020). LncRNAs are usually defined as transcripts that are longer than 200 nt in length and do not encode a discernable protein (Chekanova, 2015). Like the mRNA, lncRNAs are transcribed by Pol II, capped in their 5’ termini and poly-adenylated in their 3’ termini, and are accumulated in the cytoplasm (van Heesch et al., 2014). Ribosome profiling analyses of six species, including *Arabidopsis*, revealed that a large fraction of the lncRNAs are associated with ribosome protection (Ruiz-Orera et al., 2014). However, most of the known peptides translated from lncRNA were mostly investigated in an animal cell such as HOXB-AS3, a conserved 53-a.a. peptide encoded by lncRNA *HOXB-AS3* that could suppress colon cancer growth (Huang et al., 2017). In *Arabidopsis*, the *POLARIS* (*PLS*) gene encoding a predicted peptide of 36-a.a. residues induced by auxin was located at a 500-nt position of this transcript. PLS is required to regulate auxin–cytokinin homeostasis for modulating root growth and leaf vascular patterning (Casson et al., 2002). In legumes, ENOD40 is expressed in root nodule organogenesis. Unlike canonical mRNA, *ENOD40* is polycistronic RNA that encodes two ORFs and generates two small peptides with 12- and 24-a.a residues responsible for binding to sucrose synthase (Röhrig et al., 2002). These examples suggest that the presence of ORFs encoding for peptides in known lncORFs exists in plants; however, conclusive pieces of evidence require further investigation.

Circular RNAs (circRNAs) are produced by precursor mRNAs back-splicing where a downstream 5’ splice site is covalently linked to an upstream 3’ splice site in eukaryote (Conn et al., 2017; Li et al., 2018). In grapevine, approximately 91% of circulation events of circRNAs are exon-circulation (Gao et al., 2019), implying that circRNAs may function as a template to direct protein synthesis. In fact, the translation of some circRNAs has been discovered in animals, and the encoded peptides were found to control cell proliferation and play biological roles in disease response (Legnini et al., 2017; Shi et al., 2020). The absence of m^7^GpppN caps at the 5’ end in circRNAs and lack of poly (A) tails at the 3’ end cause cap-independent translation initiation in circRNAs (Diallo et al., 2019). Furthermore, if the circRNAs contain internal ribosome entry sites, the eukaryotic initiation factor (elF4G2) directly binds to the internal ribosome entry site and recruitments 43S pre-initiation complex to initiate translation (Wang Y. and Wang, 2015; Diallo et al., 2019; Shi et al., 2020). In humans, the N^6^-methyladenosine (m^6^A) of circRNAs drives the efficient initiation of protein translation, and even an m^6^A motif, “RRm^6^ACH” (R = G or A; H = A, C or U), is characterized (Yang et al., 2017). The coding of circRNAs has not been elucidated in plants, which potentially sheds light on another landscape.

## Regulatory Functions of Primary Microrna-Derived Peptides

**FIGURE 2:**
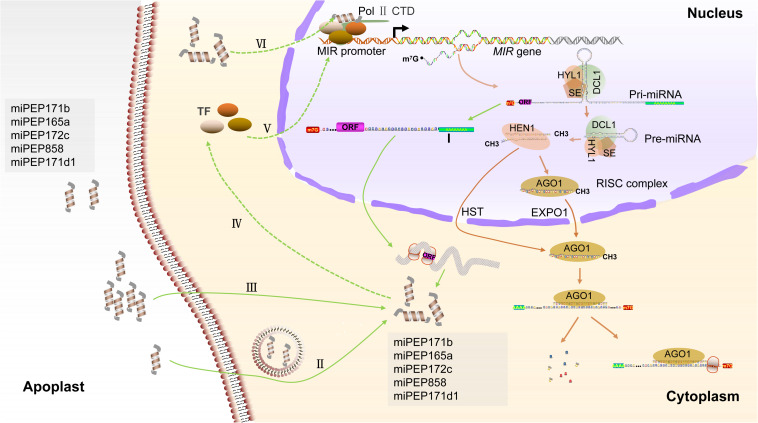
Biogenesis of miRNA and putative regulatory mechanisms of miPEPs in plants. Pri-miRNAs are transcribed by the RNA Pol II from intergenic *MIR* genes and processed by DCL complex, which is generally composed of dicer-like1 (DCL1), hyponasticleaves1 (HYL1), and serrate (SE). On the one hand, these miRNA/miRNA^∗^ duplexes that are methylated by the methyltransferase HUA enhancer1 (HEN1) and assembled into RISC in the nucleus or exported by the HST to cytoplasm for RISC assembling. RISC located in the nucleus can be exported by exportin 1 (EXPO1) to the cytoplasm (Wang et al., 2019). On the other hand, the upstream of pre-miRNA containing short ORF is possibly polyadenylated for preventing degradation (I) and is exported to cytoplasm for guiding miPEPs synthesis. External miPEPs are supposed to internalize into cytoplasm by endocytosis-associated processes (II) and passive diffusion (III) (Ormancey et al., 2020; Sharma et al., 2020). MiPEPs maybe function to transcription factors (TFs), which regulate the transcription of *MIR* genes (IV and V), resulting in upregulated expression of associated miRNA. Alternatively, miPEPs maybe serve as a part of “Pol II transcriptional complex” and enhance the transcription of *MIR* genes (VI).

Although there are modest pieces of evidence that endogenous peptides can be encoded by ncRNAs in plants, only the regulatory functions of pri-miRNA-derived peptides are well deciphered in available examples. MiRNAs are ∼22-nt regulatory elements that inhibit the expression of endogenous genes at both the transcriptional and post-transcriptional levels (Voinnet, 2009). The biogenesis of miRNAs is initialized by the Pol II-dependent transcription of intergenic *MIR* genes. Mature miRNAs are processed from the much larger pri-miRNA by the Dicer-like RNase III endonucleases (DCLs) complex and assembled into active RNA-induced silencing complex (RISC) through incorporating into ARGONAUTE1 (AGO1) protein (Rogers and Chen, 2013; Yu et al., 2017). The guide strand (miRNA) guides the RISC to bind the target gene *via* base pairing and mediates gene silencing by target cleavage or translation inhibition (Figure 2; Wang et al., 2019). In addition to producing miRNAs, it was found that the pri-miRNAs can contain short ORFs in the 5’ upstream of pre-miRNA, which encode for regulatory peptides, called miPEPs. This coding ability of pri-miRNAs has first been discovered in *Arabidopsis* and *Medicago truncatula* and has since been studied in soybeans, grapes, and even mammalian cells (Lauressergues et al., 2015; Couzigou et al., 2016; Fang et al., 2017; Chen Q. J. et al., 2020; Sharma et al., 2020). The endogenous miPEPs have been detected by Western blotting, demonstrating significant levels of peptide accumulation (Lauressergues et al., 2015; Sharma et al., 2020; Table 1).

**TABLE 1:** Length and biological function of known miPEPs in plant^*a*^.

MiPEP name	MiPEP length (a.a.)	Species	Biological function	References
miPEP171b	20	*Medicago truncatula*	Regulation of root development	Lauressergues et al., 2015
miPEP169d	NA	*Medicago truncatula*	NA	Lauressergues et al., 2015
miPEP171e	NA	*Medicago truncatula*	NA	Lauressergues et al., 2015
MiPEP165a	18	*Arabidopsis thaliana*	Regulation of root development, inflorescence stem, and flowering time	Lauressergues et al., 2015; Ormancey et al., 2020
miPEP160b	24	*Arabidopsis thaliana*	NA	Lauressergues et al., 2015
miPEP164a	37	*Arabidopsis thaliana*	NA	Lauressergues et al., 2015
miPEP319a	50	*Arabidopsis thaliana*	NA	Lauressergues et al., 2015
miPEP858a	44	*Arabidopsis thaliana*	Controlling of flavonoid biosynthesis and development	Sharma et al., 2020
miPEP171c	10	*Arabidopsis thaliana*	Regulation of primary roots	Lauressergues et al., 2015; Chen Q. J. et al., 2020
miPEP171d1	7	*Vitis vinifera*	Regulation of adventitious root formation	Chen Q. J. et al., 2020
miPEP172c	16	*Glycine max*	Stimulating nodulation	Couzigou et al., 2016

**^*a*^Numerous putative miPEPs identified in A. thaliana are presented in a previous report (Lauressergues et al., 2015).**

The mature miRNA processing mainly finishes in the nucleus and export to the cytoplasm (Wang et al., 2019). Firstly, the pri-miRNAs could be cut by the DCL complex into three parts in the nucleus: (1) the upstream of a precursor of miRNAs (pre-miRNAs), (2) the pre-miRNAs, and (3) the 3’ fragments containing poly (A) tail. The pre-miRNAs are further processed into mature miRNA, and the 3’ fragments are possibly degraded (Rogers and Chen, 2013). It is also reasonably speculated that the upstream of pre-miRNAs, which possibly contain sORF, is exported to the cytoplasm for guiding peptide translation (Figure 2). For example, the pri-miR171d is mainly accumulated in the nucleus and also slightly detected in the cytoplasm of grape, implying that the coding region of pri-miRNA is possibly transported into the cytoplasm after they are cleaved (Lauressergues et al., 2015; Chen Q. J. et al., 2020). The possibility that the cleaved upstream fragments of the pre-miRNAs are protected by re-polyadenylation is still unknown and requires investigation (Figure 2). What’s more, coupling transcription and translation in nuclear are presumed to be an alternative hypothesis (Prasad et al., 2020). The current researches suggest that plant miPEPs may serve as an endogenous peptide to positively amplify the autoregulatory feedback loop of miRNA generation (Ormancey et al., 2020). They are supposed to specifically activate transcription of their corresponding pri-miRNAs and subsequently to upregulate the expression of mature miRNAs and, meanwhile, to enhance the accumulation of their own levels. The positive regulatory function of miPEPs on *MIR* transcription in the plant has been investigated. In *Arabidopsis*, the positive effect of miPEP165a on pri-miR165a accumulation is inhibited by the cordycepin, which is an inhibitor of RNA synthesis (Lauressergues et al., 2015). In *Arabidopsis*, the promotor of miR858a normally activates the expression of the *GUS* gene in two reporter lines (PromiR858a:ATG^1^:GUS and PromiR858a:ORF^1^:GUS), which is fused only the start code or entire ORF encoded miPEP858a. Furthermore, the GUS activity is enhanced by the supplement with synthetic miPEP858a in the media, indicating that the miPEP858a acts on the promotor region for enhancing transcription (Sharma et al., 2020). On the one hand, miPEPs possibly, directly or indirectly, function as a *trans*-acting factor such as a transcription factor (TF), which positively regulates the transcription of *MIR* genes (Figure 2). Conventional peptides can regulate TF expression levels in the plant. Root meristem growth factor 1 is a secreted and Tyr sulfated peptide and required to maintain the root stem cell niche and transit amplifying cell proliferation in *Arabidopsis*. Root meristem growth factor 1 positively regulates the expression levels of PLT, which is a root-specific TF mediating pattern of the root stem cell niche (Matsuzaki et al., 2010). On the other hand, miPEPs may bind one of the subunits of “Pol II transcriptional complexes” or bind to the Pol II, although there are no direct pieces of evidence to support this hypothesis (Figure 2). In plants, peptides can also interact with the subunit of catalyzing enzyme. A representative example is ENOD40 peptides in legumes. Two overlapped ORFs located in 5′ conserved region encode two peptides of 12- and 24-a.a. length residues (peptides A and B); both peptides can specifically bind to nodulin 100 that is a subunit of sucrose synthase (Röhrig et al., 2002). In a word, the strictly regulatory mechanisms of miPEPs remain elusive.

Interestingly, the external application of synthetic miPEPs, which probably do not need additional modification and processing, to plants can produce the same autoregulatory effect. Unlike precursor-derived peptides that usually act as ligand recognized by associated receptors, miPEPs are hypothesized to be internalized by passive diffusion and endocytosis-associated processes (Kachroo et al., 2001; Okuda et al., 2009; Yamaguchi et al., 2010; Oh et al., 2018; Ormancey et al., 2020). In *Arabidopsis*, fluorescently labeled miPEP165a rapidly penetrates into the whole root during 24 h. Loss of function of genes associated with endocytosis or application of endocytosis inhibitor influences the uptake of miPEP165a in the meristematic zone and differentiation zone (Ormancey et al., 2020). Similar to observations of miPEP165a, miPEP858a can be absorbed by the roots and presence inside the plant cell (Sharma et al., 2020). These results indirectly provide pieces of evidence that miPEPs possibly play a role for regulatory functions within intracellular space rather than be transported into the apoplast (Figure 2).

An investigation of 50 *Arabidopsis* pri-miRNAs uncovers the presence of at least one putative ORF encoding miPEPs in one pri-miRNA (Lauressergues et al., 2015). These miPEPs have no common signatures, implying that each of these miPEPs is likely specific for their miRNA (Lauressergues et al., 2015). Such a large number of miPEPs form a complex and specific regulatory network that performs different biological functions by positively regulating miRNA expression level. So far, the biological function of numerable miPEPs has been deciphered. MiPEP171b and miPEP165a are 20-a.a. and 18-a.a. peptides produced by *M. truncatula* and *Arabidopsis*, respectively. Overexpression and exogenous supplement of these peptides specifically trigger the accumulation of *miR171b* and *miR165a*, resulting in decreased lateral root formation and stimulation of main root growth (Lauressergues et al., 2015). Watering plants with synthetic miPEP172c increases nodule number in soybean (Couzigou et al., 2016). In grape, an exogenous supplement of vvimiPEP171d1 can promote adventitious root development by enhancing the expression of *vvi-MIR171d* (Chen Q. J. et al., 2020). MiPEP858a is a 44-a.a. peptide encoded by the first ORF (135 bp) located upstream in the pre-miR858a sequence in *Arabidopsis*. The endogenous miPEP858a is ∼6 kDa in molecular weight (Sharma et al., 2020). MiPEP858a controls flavonoid biosynthesis and plant development by regulating the expression of genes involved in the phenylpropanoid pathway and auxin signaling (Sharma et al., 2020). Although the regulatory functions of several miPEPs have been experimentally validated in different plant species, some key questions remain to be answered. Is miPEPs specific for upregulation of their corresponding pri-miRNAs, and if so, how is it achieved? After all, only a few miRNAs have been used to detect activation specificity (Lauressergues et al., 2015). In grape, the expression level of some miRNAs genes such as *vvi-MIR160c*, *vviMIR171a*, and *vvi-MIR171i* even reduces when grape tissue culture plantlets are treated by synthetic vvi-miPEP171d1, even if it is not definitely clear whether this decrease is caused by the incubation period (Chen Q. J. et al., 2020). In fact, small peptides encoded by lncRNAs produce either inhibitory or stimulatory effects on their target genes in mammals (Anderson et al., 2015; Nelson et al., 2016). Therefore, whether miPEPs exert a negative effect on their corresponding miRNAs or other miRNAs remains to be shown.

## Perspective

Peptides are regulatory molecules that have received great attention over recent years. In particular, different types of peptides identified from several species were found to be enriched in the peptidome of plants (Tavormina et al., 2015; Olsson et al., 2019; Takahashi et al., 2019). In addition to the conventional peptides derived from the precursor processing and short ORFs, pieces of evidence are emerging for the presence and function of non-conventional peptides translated from 5’ UTR or 3’ UTR of transcripts and currently defined as ncRNAs (Wang et al., 2020). The function of peptides is diverse, and they are found to be involved in development, growth, and reproduction, senescence and cell death, nutrients balance and nodulation, and biotic and abiotic stress responses. In the past few years, numerous miPEPs derived from pri-miRNAs have been experimentally identified and their function suggested by overexpression studies in different species, implying that coding of pri-miRNAs is ubiquitous among different plant species. Existing pieces of evidence support the regulatory ability of miPEPs in directing an increase in the level of their associated miRNA by enhancing the transcription of pri-miRNAs. Still, several questions remain to be addressed. (1) MiPEPs synthesis and miRNA processing occur in two independent regions of pri-miRNAs; how does coordination occur between the translation in the cytoplasm and the maturation of miRNA in the nucleus? One putative mechanism is that the 5′ upstream of pri-miRNA is transported into the cytoplasm for guiding miPEP synthesis after releasing from the dicing complex and re-polyadenylation (Figure 2). (2) How do miPEPs specifically enhance transcription of the *MIR* gene? An analysis of 50 *Arabidopsis* miPEPs uncovered no common structural pattern among them, suggesting that each miPEP probably has a specific regulatory function (Lauressergues et al., 2015). Whether miPEPs directly bind Pol II or trans-acting factors such as TFs remains to be further investigated. (3) Regarding the exogenous application of miPEPs, overexpression and CRISPR-Cas9-editing are required for assessing the biological functions. It is not clear that post-translational modifications of miPEPs can enhance the activity of miPEPs.

In summary, most characterized peptides to date are hypothesized to act as a ligand to mediate plant intercellular communication and response. The identification of peptides that are translated from the transcript of currently defined ncRNAs enriches the plants’ peptidome. Particularly, miPEP identification uncovers the dual function of pri-miRNAs combing with coding and non-coding ability. Dissecting the biosynthesis and regulatory mechanism of miPEPs will reveal another miRNA-dependent gene regulation network.

## Author Contributions

YR integrated the manuscript and drafted the figures. YS, LZ, DG, and JH retrieved and collected the references about miRNA biogenesis, translation of circRNAs, and the translation of lncRNA. LW, SS, WX, and CZ retrieved and collected the literatures about canonical peptides biogenesis, and biological functions. AL and SW revised the manuscript. CM conceived the idea and revised the manuscript. All authors contributed to the article and approved the submitted version.

## Conflict of Interest

The authors declare that the research was conducted in the absence of any commercial or financial relationships that could be construed as a potential conflict of interest.
